# Phenotypic and genomic relationships between vulva score categories and reproductive performance in first-parity sows

**DOI:** 10.1186/s40104-020-00527-1

**Published:** 2021-01-12

**Authors:** Flor-Anita Corredor, Leticia P. Sanglard, Jason W. Ross, Aileen F. Keating, Richard J. Leach, Nick V. L. Serão

**Affiliations:** 1 Department of Animal Science, Iowa State University, Ames, IA 50011 USA; 2 Iowa Pork Industry Center, Iowa State University, Ames, IA 50011 USA

**Keywords:** Genetic parameters, GWAS, Reproduction, Swine, Vulva

## Abstract

**Background:**

One of the biggest challenges in the swine industry is to increase female reproductive efficiency. Recently, vulva score categories (VSC), assessed prior to puberty, has been proposed as an indicator trait of efficient reproductive performance in sows. The objective of this study was to validate the use of VSC as an indicator trait for reproductive performance, and to perform genetic and genomic analyses for VSC.

**Methods:**

The phenotypic relationship of VSC, using a three-point scale: small (VSC-S), medium (VSC-M), and large (VSC-L), on reproductive performance was evaluated on three farms. VSC was measured at 15 weeks of age, for farms 1 and 2, and at 14 weeks of age for farm 3 on 3981 Yorkshire gilts, in which 1083 had genotypes (~ 50 K SNPs). Genetic parameters for VSC with reproductive traits were estimated using ssGBLUP. A Genome-wide association study (GWAS) for VSC was performed using BayesB.

**Results:**

For the phenotypic analysis of VSC across datasets, differences in performance were identified there was a significant effect (*P* ≤ 0.05) for the interaction between Farm and VSC for total number dead (TND), and a trend (*P* < 0.10) for total number born (TNB). There were significant (P ≤ 0.05) pre-defined contrasts of VSC-S versus VSC-M + L on TNB, number born alive (NBA), TND, number of stillborn (NSB), and number of mummies (MUM). Heritability estimates for VSC as a categorical trait (VSCc) and a quantitative trait (VSCq) were 0.40 ± 0.02 and 0.83 ± 0.02, respectively, for across farm, 0.13 ± 0.07 and 0.20 ± 0.10, respectively, for Farm1, 0.07 ± 0.07 and 0.09 ± 0.09, respectively, for Farm2, and 0.20 ± 0.03 and 0.34 ± 0.05, respectively, for Farm3. For across farms, favorable genetic correlations estimates were found for TNB (0.28 ± 0.19) and NBA (0.26 ± 0.17). Within farms, moderate genetic correlations between VSC with reproductive traits were found for TNB (0.61 ± 0.47) and MUM (0.69 ± 0.47) for farm 1, for number of services until first farrow (NS; 0.69 ± 0.38) and unique service with successful first farrow (SFS; − 0.71 ± 0.38) for farm 3. Multiple genomic regions associated with VSC_c_ were identified. Of these, a QTL located on chromosome 3 at 33–34 Mb accounted for about 7.1% of the genetic variance for VSC_c_ and VSC_q_. This region harbors the gene *PRM1* that has been associated with early embryonic development in pigs.

**Conclusions:**

The results support potential of VSC for improved reproductive efficiency on first-parity performance, but the results might depend on the interaction between environmental factors and VSC, as well as potentially additive genetics.

## Background

The efficiency of reproductive performance in the swine industry is critical to maximizing productivity. However, genetic selection for reproductive traits in sows (e.g., litter size traits) is challenging because of their low heritability [1]. Additional difficulties for genetic selection for these traits include the fact that these traits are sex-dependent and expressed later in life. To overcome these limitations, one strategy could be the identification of an indicator trait, which should have: high heritability, have high favorable genetic correlation (*r*_*G*_) with reproductive traits, be easy and cheap to measure, and be expressed early in life. Recently, the use of vulva size as an indicator trait for reproductive traits has been explored [2, 3]. Pre-pubertal gilts with larger vulva width at ~ 15 weeks of age had greater follicular activity and reached puberty at a younger age compared to those with smaller vulva width [2]. Romoser et al. [3] reported favorable phenotypic relationship between vulva width scores and litter size in sows, suggesting that vulva score categories (VSC) could be used as a proxy for reproductive performance. Gilts classified as having large VSC had higher first farrowing rates (84.4% vs. 64.7%) and number of piglets born at first parity (12.4 vs. 11.8) compared to gilts classified as having small VSC [2]. On the genetic side, knowledge is scant regarding this novel trait. Knauer et al. [4] explored the genetics of vulva width in gilts of approximately 162 days of age and reported a moderate heritability estimated (*h*^2^ = 0.57). Corredor et al. [5] reported genetic parameters and QTL for vulva size traits in Landrace and Yorkshire gilts. Heritability estimates in Yorkshire gilts ranging from 0.31 (vulva width) to 0.55 (vulva height), and major QTL for VS on chromosomes 1 (87–91 Mb and 282–287 Mb), and 5 (67 Mb) were observed, explaining up to 6.9% of the genetic variance [5]. However, results from Romoser et al. [3] have not been validated in an independent dataset and no studies, to the best of our knowledge, have evaluated both genetic and phenotypic relationships between VSC and reproductive traits. Therefore, the objectives of this study were to 1) validate the phenotypic relationship of VSC on reproductive performance, validating the work of Romoser et al. [3] using an additional datasets, 2) estimate genetic parameters for VSC and reproductive traits; and 3) perform GWAS for VSC in three populations of first-parity gilts.

## Methods

### Animals and phenotype data

A total of 3981 (Farm1 + 2 + 3) Yorkshire gilts from three farms located in Colorado, USA, were used in this study, with 746, 722, and 2513 from farms 1 (Farm1), 2 (Farm2), and 3 (Farm3). All animals were from the same genetic source and were reared under the same controlled conditions. At 15 weeks of age in Farm1 and Farm2, and at 14 weeks of age in Farm3, all gilts were assigned a VSC, following methodology described in Romoser et al. [3]. The VSC of gilts were visually categorized by the same trained person in the three farms using a three-point scale: small (VSC-S), medium (VSC-M), and large (VSC-L). No additional information was available for these farms. The frequencies of the VSC for each farm were 21, 547, and 178, respectively, for Farm1; 12, 533, and 177, respectively, for Farm2; and 532, 1569, and 412, respectively, for Farm3 (Table 1).

**Table 1:** Number of individuals with phenotype, genotype, and vulva score categories (VSC^a^) information per farm

Data	Farm1	Farm2	Farm3	Total
Phenotype	746	722	2513	3981
Genotype	193	202	688	1083
VSC-S	21	12	532	565
VSC-M	547	533	1569	2649
VSC-L	178	177	412	767

^a^Vulva score categories: small (VSC-S), medium (VSC-M), and large (VSC-L)

First-parity performance traits included number of piglets born alive (NBA), number of stillborn piglets (NSB), and number of mummified piglets (MUM). Total number of born dead piglets (TND) was calculated as NSB + MUM, and total number of born piglets (TNB) was calculated as NBA + TND. Prior to statistical analyses, data on TND, NSB, and MUM were transformed in order to meet the assumptions of statistical inference [6] using ln(*y* + 1), where *y* represents the observed phenotype of the trait. In addition, farrowing traits included: age at first service (AFS) in days, number of services until first farrow (NS), and unique service with successful first farrow (SFS, success = 1, fail = 0). In this study, a single service was defined as any service events within 14 days. Therefore, a second service event was any one occurring 14 days after the first event. The summary statistics of these traits is shown on Table 2. A 9-generation pedigree including 9080 individuals was available.

**Table 2:** Summary statistics

Trait^a^	Mean	SD	Min	Max
Farm1 (*n* = 746)				
AFS	224.5	13.1	199	277
TNB	11.4	2.7	4	20
NBA	10.1	2.7	0	18
TND	1.2	1.7	0	14
NSB	0.6	1.1	0	13
MUM	0.6	1.1	0	8
NS	1.1	0.2	1	2
SFS	0.9	0.2	0	1
Farm2 (*n* = 722)				
AFS	226.0	12.9	197	272
TNB	11.4	2.8	4	19
NBA	10.5	2.9	0	18
TND	0.9	1.5	0	12
NSB	0.5	0.9	0	6
MUM	0.4	1.0	0	8
NS	1.1	0.2	1	2
SFS	0.9	0.2	0	1
Farm3 (*n* = 2513)				
AFS	235.6	15.1	187	360
TNB	12.0	3.0	4	25
NBA	11.3	2.9	0	20
TND	0.7	1.3	0	16
NSB	0.5	1.0	0	16
MUM	0.2	0.7	0	8
NS	1.1	0.3	1	4
SFS	0.9	0.3	0	1

^a^*AFS* Age at first service, *TNB* Total number born, *NBA* Number born alive, *TND* Total number dead, *NSB* Number stillborn, *MUM* Number of mummies, *NS* Number of services until first farrow *SFS* Unique service with successful first farrow (Median = 1 for all farms)

### Genotype data

Genotype data were available for 193, 202, and 688 animals for Farm1, Farm2, and Farm3, respectively (Table 1). DNA was isolated from tail or ear tissue using the ReliaPrep 96/KingFisher tissue kits (Promega, Madison, WI, USA). Individuals were genotyped using a custom Affymetrix/Thermo Fisher Axiom® genotyping array containing 51,467 evenly spaced SNPs. Markers without known position, located on sexual chromosomes, with minor allele frequencies below 0.01, and with a call rate below 0.8 were excluded. After this genotype quality control performed on the genotypic information from all farms combined, a total of 6778 SNPs were excluded. The final number of SNPs that remained in the data set were 44,689 SNPs. The remaining missing SNP genotypes were imputed chromosome-wise across all farms genotype information combined using a Hidden Markov Model based algorithm implemented in Eagle v.2.4.1 software [7]. A previous study using other animals from the same population has shown an imputation accuracy of missing genotypes of over 95% using this SNP chip [8].

### Phenotypic analysis of vulva score categories on reproductive traits

The phenotypic relationship between VSC with reproductive traits (for TNB, NBA, TND, and MUM) was evaluated using the single-step BLUP (ssGBLUP) procedure [9] in the following model:

1 $$ {y}_{ij k}=\mu +{Farm}_i+{VSC}_j+{\left( Farm\times VSC\right)}_{ij}+{a}_k+{e}_{ij k} $$

Where *y*_*ijk*_ is the observed phenotype (i.e., reproductive traits); *μ* is the general mean; *Farm*_*i*_ is the *i*^th^ level of the fixed-effect of farm; *VSC*_*j*_ is the *j*^th^ level of the fixed-effect of vulva score category; (*Farm* × *VSC*)_*ij*_ is the interaction term between *Farm*_*i*_ and *VSC*_*j*_; *a*_*k*_ is the animal random effect of the *k*^th^ animal, assuming ${a}_k\sim N\left(0,\boldsymbol{H}{\sigma}_a^2\right)$, where ***H*** is the additive genetic relationship matrix including genotyped and non-genotyped animals [10]; and *e*_*ijk*_ is the random error term associated with *y*_*ijk*_, assuming ${e}_{ijk}\sim N\left(0,\boldsymbol{I}{\sigma}_e^2\right)$, where ***I*** is the identity matrix. For TNB, NBA, TND, NSB, and MUM, the effect of VSC was estimated using a similar model, with the addition of a random effect of contemporary group (CG, combination of year and week of farrow), assuming ${CG}_l\sim N\left(0,\boldsymbol{I}{\sigma}_{CG}^2\right)$. For NS, the random effect of CG was included in the model as a combination of year and week of service. For the analysis of NS and SFS, the effect of AFS was included as a covariate in order to account for the age of the gilt at time of insemination. In addition to testing the overall effect of VSC on reproductive traits, an additional contrast was evaluated following Romoser et al. [3], in which we tested the difference between VSC-S and the average of VSC-M and VSC-L, as well as of another contrast comparing the average of VSC-S and VSC-M with VSC-L. The threshold for significant and trending effects were *P*-value< 0.05 and *P*-value< 0.10, respectively. In addition, we also evaluated the effect of random effect of service sire on these models. However, due to the high number of missing data and lack of effect (i.e., < 1% of the variation explained by this effect), this strategy was not further pursued. All analyses were performed in ASReml v4.0 [11].

### Genetic parameters and efficiency of correlated response to selection

Genetic parameters for VSC were estimated using the following animal model:

2 $$ {y}_{ij}=\mu +{Farm}_i+{a}_j+{e}_{ij} $$

Where *y*_*ij*_ is the observed phenotype; *μ* is the overall mean; *Farm*_*i*_ is the *i*^th^ level of the fixed-effect of farm; *a*_*j*_ is the animal random effect of the *j*^th^ animal, assuming ${a}_j\sim N\left(0,\boldsymbol{H}{\sigma}_a^2\right)$, where ***H*** is the additive genetic relationship matrix including genotyped and non-genotyped animals [10]; and *e*_*i*_ is the random error term associated with *y*_*ij*_, assuming ${e}_{ij}\sim N\left(0,\boldsymbol{I}{\sigma}_e^2\right)$. In addition to this model, we had also evaluated the random effects of week of VSC measurement and common-environment (i.e., litter effect) in the model, but the variance estimates for these effects were close to zero (data not shown), and hence, these effects were not included in the final model. Genetic parameters for AFS, TNB, NBA, TND, NSB, MUM, NS, and SFS were estimated including the appropriate random CG effect, as previously described in model (1). In addition to the model described above for all three farms, these analyses were also performed for each farm separately. Genetic parameters were estimated for VSC as a categorical (VSC_c_) and as a continuous (VSC_q_) trait. Heritabilities were estimated using a probit mixed model for VSC_c_ and SFS, and using a general mixed linear model for all other traits. Genetic and phenotypic correlations were estimated within farm between VSC and reproductive traits, and genetic correlations between farms for VSC. Due to software limitations, these were estimated for VSC_q_. The efficiency (E) of correlated response to selection was estimated as:

3 $$ E={r}_G\left(\raisebox{1ex}{${h}_{VSC}$}\!\left/ \!\raisebox{-1ex}{${h}_{Trait\ of\ interest}$}\right.\right) $$

Where *r*_*G*_ is the estimated genetic correlation between VSC and the reproductive trait of interest; *h*_*VSC*_ is the square root of the heritability estimate for VSC; and *h*_*Trait of interest*_ is the square root of the heritability estimate for a reproductive trait of interest.

### Genome-wide association analysis

Genome-wide association studies (GWAS) was performed for VSC for each farm and across farms, using Bayesian genomic prediction methods [12] using the following model:

4 $$ {y}_i=\mu +\sum \limits_{i=1}^j{m}_{ij}{\upalpha}_j+{e}_i $$

Where *y*_*i*_ is the observed phenotype; *μ* is the overall mean; *m*_*ij*_ is the genotype at the *j*^th^ SNP for the animal *i*; *a*_*j*_ is the allele substitution effect for the *j*^th^ SNP, and *e*_*i*_ is the error term associated with *y*_*i*_, assuming ${e}_i\sim N\left(0,\boldsymbol{I}{\sigma}_e^2\right)$. Additionally, data on the three farms were analyzed simultaneously, and in this analysis, the fixed effect of farm was included in the model. The estimates of additive genetic and residual variances obtained from the genetic parameter estimation were used as priors in BayesC analysis, assuming all SNPs with an effect (i.e., π = 0). Then, BayesCπ was performed to estimate the proportion of SNPs with zero effect (π). Afterwards, analyses were performed using BayesB, with a π = 0.999. Analyses were carried out using 50,000 iterations using Gibbs sampling, and a burn-in of 5000 cycles. Analyses were performed in GenSel version 4.4 [13].

Putative candidate genes within identified QTL regions and in the neighboring upstream and downstream 3-Mb regions were identified based on the Sscrofa11.1 genome assembly, using the BioMart tool from the Ensembl Genome Browser (https://www.ensembl.org/index.html). The 3-Mb neighboring regions of each side of the identified regions were investigated to account for the resolution of the QTL mapping method used in this study [14]. QTL regions explaining at least 1% of the total genetic variance accounted for by the markers (TGVM) were discussed in this study, including the identification of candidate genes within these QTL.

## Results

### Phenotypic analysis of vulva score categories on reproductive traits

The phenotypic relationship of VSC on reproduction performance is shown in Table 3. For the phenotypic analysis of VSC across datasets (Table 3), there was a significant effect (*P* ≤ 0.05) for the interaction between Farm and VSC for TND, and a trend (*P* < 0.10) for TNB. Although this interaction was not significant (*P* ≥ 0.11) for NBA, NSB, and MUM, we observed a significant (*P* ≤ 0.05) pre-defined contrast of VSC-S versus M + L for these traits, as well as for TNB, and TND.

**Table 3:** Effect of vulva score categories (VSC) on reproductive traits across datasets

Trait^1,2^	Farm	VSC^3^			*P*-value^4^				
		S	M	L	Farm	VSC	Farm×VSC	S vs. M + L	S + M vs. L
AFS	1	220.67 (3.14)	224.52 (0.61)	224.9 (1.08)	< 0.01	0.89	0.72	0.24	0.73
	2	224.5 (4.15)	226.19 (0.62)	225.55 (1.08)					
	3	235.59 (0.62)	235.55 (0.36)	235.78 (0.71)					
TNB	1	10.34 (0.65)	11.63 (0.15)	11.51 (0.23)	< 0.01	0.08	0.06	< 0.01	0.56
	2	13.13^a^ (0.85)	11.34^b^ (0.15)	11.52^b^ (0.23)					
	3	11.69^b^ (0.14)	11.99^a^ (0.10)	12.12^a^ (0.16)					
NBA	1	9.7^b^ (0.63)	10.21^a^ (0.13)	10.26^a^ (0.22)	< 0.01	0.03	0.33	0.04	0.69
	2	11.8 (0.83)	10.43 (0.13)	10.56 (0.22)					
	3	10.89^b^ (0.13)	11.28^a^ (0.08)	11.46^a^ (0.15)					
TND	1	0.35^b^ (0.05)	0.86 ^a^ (0.01)	0.78^a^ (0.02)	< 0.01	0.90	0.05	0.01	0.50
	2	0.82^a^ (0.07)	0.55^b^ (0.01)	0.62^a^ (0.02)					
	3	0.48 (0.01)	0.45 (0.01)	0.45 (0.01)					
NSB	1	0.17^b^ (0.04)	0.45^a^ (0.01)	0.35^a^ (0.02)	< 0.01	0.99	0.11	0.05	0.31
	2	0.55^a^ (0.06)	0.29^b^ (0.01)	0.35^b^ (0.02)					
	3	0.35 (0.01)	0.32 (0.01)	0.32 (0.01)					
MUM	1	0.15^b^ (0.04)	0.41^a^ (0.01)	0.41^a^ (0.01)	< 0.01	0.24	0.20	0.04	0.33
	2	0.26 (0.05)	0.26 (0.01)	0.23 (0.01)					
	3	0.15 (0.01)	0.15 (0.01)	0.10 (0.01)					
NS	1	1.00^AB^ (0.07)	1.04^A^ (0.01)	1.07^B^ (0.02)	< 0.01	0.08	0.58	0.55	0.21
	2	1.08^AB^ (0.09)	1.05^A^ (0.01)	1.04^B^ (0.02)					
	3	1.10^AB^ (0.01)	1.11^A^ (0.01)	1.14^B^ (0.02)					
SFS	1	1.01^A^ (0.06)	0.96^AB^ (0.01)	0.93^B^ (0.02)	< 0.01	0.05	0.60	0.59	0.23
	2	0.92^A^ (0.08)	0.95^AB^ (0.01)	0.96^B^ (0.02)					
	3	0.92^A^ (0.01)	0.91^AB^ (0.01)	0.87^B^ (0.01)					

^1^AFS: Age at first service, TNB: Total number born, NBA: Number born alive, TND: Total number dead, NSB: Number stillborn, MUM: Number of mummies, NS: Number of services until first farrow, SFS: Unique service with successful first farrow

^2^Means after back log-transformation are show for TND, NSB, MUM

^3^Vulva score categories: small (S), medium (M), and large (L)

^4^Pre-defined contrasts. S vs. M + L tested the difference between VSC-S against the average of VSC-M with VSC-L, whereas S + M vs. L tested the difference between the average of VSC-S with VSC-M against VSC-L

^a-b^Means lacking the same superscript within a row are significantly different at *P* < 0.05 based on Farm×VSC or pre-defined contrast

^A-B^Means lacking the same superscript within a trait are significantly different at *P* < 0.05 based on the main effect of VSC

The phenotypic relationship between VSC and reproductive traits diverged among farms. For TNB, although VSC-S had greater (*P* < 0.05) TNB (13.13 ± 0.85) than VSC-M and VSC-L (11.43 ± 0.19) in Farm2, the relationship in Farm3 was opposite; VSC-S gilts had fewer (*P* < 0.05) TNB (11.69 ± 0.14) compared to the VSC-M and VSC-L (12.06 ± 0.13) gilts. No relationships were found (*P* > 0.05) between VSC and TNB on Farm1. For NBA, for Farm3, the same relationship found for TNB was observed for this trait, with greater (*P* < 0.05) performance in VSC-M and VSC-L compared to VSC-S. In contrast, there was no relationships found for Farm2 (*P* > 0.05), whereas in Farm1, VSC-S had the lower (*P* < 0.05) performance (9.70 ± 0.63) than VSC-M and VSC-L (10.24 ± 0.18).

The relationships found for TND were more complex. For Farm1, the relationship was the same as for NBA, with lower (*P* < 0.05) number of piglets in VSC-S gilts (0.35 ± 0.05) compared to VSC-M and VSC-L (0.82 ± 0.02). For Farm2, VSC-M had the fewer (*P* < 0.05) piglets (0.55 ± 0.01) than VSC-S and VSC-L (0.72 ± 0.05), whereas no relationships were found for Farm3 (*P* < 0.05). For NSB, the same relationships found for TND were found, with greater performance found in VSC-S gilts on Farm1, and lower performance found in VSC-S gilts of Farm2 compared to VSC-M and VSC-L (*P* < 0.05 for both farms). No relationships were found (*P* > 0.05) between VSC and NSB on Farm3. Finally, VSC-S gilts showed fewer (*P* < 0.05) MUM (0.15 ± 0.04) than VSC-M and VSC-L (0.41 ± 0.01) on Farm1, whereas no relationships (*P* > 0.05) were found for the other farms. These results show that the overall relationship between VSC and reproductive performance depends on the environment (i.e., farm) in which these gilts are raised in.

We also found overall relationships between VSC and reproductive performance. There were relationships between VSC with NS (*P* = 0.08), and SFS (*P* = 0.05), with VSC-S showing overall greater performance than VSC-M and VSC-L gilts. VSC-S required fewer (*P* < 0.05) NS (1.06 ± 0.04) than VSC-L gilts (1.08 ± 0.01) and had greater (*P* < 0.05) SFS (0.94 ± 0.03) than VSC-L gilts (0.92 ± 0.01). Although we found a trend effect of Farm×VSC on TNB (*P* = 0.06) and significant (*P* < 0.01) pre-defined contrast (S vs. M + L), there was a trend (*P* = 0.08) for the main effect of VSC, with VSC-M (11.65 ± 0.09) having lower performance (*P* < 0.05) than VSC-S (11.72 ± 0.36) and VSC-L (11.72 ± 0.13). Finally, there was an effect of Farm for all traits analyzed (*P* < 0.01), indicating that the environment is a major contributor for the variability of the reproductive data in this study.

### Genetic parameters and efficiency of correlated response to selection

Estimates of heritability are presented in Table 4. Heritability estimates for VSC_c_ and VSC_q_ were 0.40 ± 0.02 and 0.83 ± 0.02, respectively, for across farm, 0.13 ± 0.07 and 0.20 ± 0.10, respectively, for Farm1, 0.07 ± 0.07 and 0.09 ± 0.09, respectively, for Farm2, and 0.20 ± 0.03 and 0.34 ± 0.05, respectively, for Farm3. For reproductive traits, the across farm dataset presented low heritability estimates ranging from < 0.01 ± < 0.01 (MUM) to 0.08 ± 0.03 (AFS). Farm1 presented moderate heritability estimates for AFS (0.37 ± 0.12), whereas estimates were low for the remaining traits, ranging from < 0.01 ± 0.01 (SFS) to 0.13 ± 0.08 (NBA). For Farm2, heritability estimates for AFS, TND, and NSB were moderate, with 0.27 ± 0.11, 0.28 ± 0.10, and 0.27 ± 0.09, respectively. Heritability estimates for the remaining traits were low, ranging from 0.03 ± 0.07 (NBA) to 0.17 ± 0.11 (SFS). For Farm3, heritability estimates were low, ranging from 0.02 ± 0.03 (TND) to 0.14 ± 0.04 (AFS). For VSC_q_, estimates of residual variances (${\sigma}_e^2$) were lower for across farms and somewhat similar for the three farms. Estimates of additive genetic variances (${\sigma}_a^2$) for VSC_q_ for across farms was substantially greater (0.30) when compared to the subset dataset by farms (ranging from 0.02 to 0.13). Estimates of *r*_*G*_ for VSC_q_ between farms were high for all comparisons, with estimates of 0.97 ± 0.25 (Farm1 and Farm2), 0.77 ± 0.22 (Farm1 and Farm3), and 0.98 ± 0.16 (Farm2 and Farm3).

**Table 4:** Estimates (standard errors in parentheses) of residual (${\sigma}_e^2$) and additive genetic (${\sigma}_a^2$) variances, and heritability (*h*^2^**)**

	Farm1 + 2 + 3			Farm1			Farm2			Farm3		
Trait^a^	${\boldsymbol{\sigma}}_{\boldsymbol{e}}^{\boldsymbol{2}}$	${\boldsymbol{\sigma}}_{\boldsymbol{a}}^{\boldsymbol{2}}$	*h*^2^	${\boldsymbol{\sigma}}_{\boldsymbol{e}}^{\boldsymbol{2}}$	${\boldsymbol{\sigma}}_{\boldsymbol{a}}^{\boldsymbol{2}}$	*h*^2^	${\boldsymbol{\sigma}}_{\boldsymbol{e}}^{\boldsymbol{2}}$	${\boldsymbol{\sigma}}_{\boldsymbol{a}}^{\boldsymbol{2}}$	*h*^2^	${\boldsymbol{\sigma}}_{\boldsymbol{e}}^{\boldsymbol{2}}$	${\boldsymbol{\sigma}}_{\boldsymbol{a}}^{\boldsymbol{2}}$	*h*^2^
VSC_c_	1.00	0.66	0.40 (0.02)	1.00	0.15	0.13 (0.07)	1.00	0.07	0.07 (0.07)	1.00	0.25	0.20 (0.03)
VSC_q_	0.06	0.30	0.83 (0.02)	0.19	0.05	0.20 (0.10)	0.19	0.02	0.09 (0.09)	0.25	0.13	0.34 (0.05)
AFS	192.14	16.39	0.08 (0.03)	116.69	69.20	0.37 (0.12)	128.53	46.40	0.27 (0.11)	201.00	32.09	0.14 (0.04)
TNB	8.29	0.17	0.02 (0.02)	6.27	0.25	0.03 (0.05)	7.27	0.81	0.10 (0.09)	8.63	0.58	0.06 (0.04)
NBA	8.18	0.03	< 0.01 (0.02)	6.10	0.99	0.13 (0.08)	8.47	0.27	0.03 (0.07)	7.55	0.85	0.10 (0.05)
TND	0.06	< 0.01	0.03 (0.02)	0.07	< 0.01	0.06 (0.06)	0.04	0.02	0.28 (0.10)	0.05	< 0.01	0.02 (0.03)
NSB	0.04	< 0.01	0.07 (0.03)	0.04	< 0.01	0.05 (0.05)	0.03	0.01	0.27 (0.09)	0.04	< 0.01	0.04 (0.03)
MUM	0.03	< 0.01	< 0.01 (< 0.01)	0.04	< 0.01	0.05 (0.06)	0.03	< 0.01	0.08 (0.08)	0.02	< 0.01	0.08 (0.06)
NS	0.09	< 0.01	0.02 (0.02)	0.05	< 0.01	< 0.01 (0.01)	0.05	< 0.01	0.08 (0.06)	0.11	< 0.01	0.03 (0.03)
SFS	1.00	0.06	0.06 (0.04)	1.00	< 0.01	< 0.01 (0.01)	1.00	0.20	0.17 (0.11)	1.00	< 0.01	0.03 (0.05)

^a^VSC_c_: Vulva score as categorical variable, VSC_q_: Vulva score as continuous variable, AFS: Age at first service, TNB: Total number born, NBA: Number born alive, TND: Total number dead, NSB: Number stillborn, MUM: Number of mummies, NS: Number of services until first farrow, SFS: Unique service with successful first farrow

Estimates of phenotypic (*r*_*P*_) and genetic (*r*_*G*_) correlations between VSC_q_ with reproductive traits for each farm are presented in Table 5. For across farms, estimates of *r*_*G*_ were low, ranging from 0.28 ± 0.19 (TNB) to − 0.30 ± 0.13 (SFS). Favorable *r*_*G*_ estimates were found for TNB (0.28 ± 0.19) and NBA (0.26 ± 0.17), whereas unfavorable estimates were found for AFS (0.25 ± 0.06), TND (0.12 ± 0.20), MUM (0.27 ± 0.79), NS (0.18 ± 0.13), and SFS (− 0.30 ± 0.13), although standard errors were overall large. For the within farm analyses, Farm1 had moderate to high estimates of *r*_*G*_ between VSC_q_ with TNB (0.61 ± 0.47), NBA (0.30 ± 0.37), MUM (0.69 ± 0.47), and SFS (− 0.36 ± 0.95). Estimates of *r*_*G*_ between VSC_q_ with the remaining traits were lower, ranging from 0.10 ± 0.55 (NSB) to 0.26 ± 0.51 (TND). For Farm2, estimates of *r*_*G*_ were moderate between VSC_q_ with AFS (− 0.51 ± 0.44), NSB (0.38 ± 0.35), MUM (− 0.42 ± 0.58), NS (− 0.31 ± 0.59), and SFS (0.33 ± 0.58). Estimates of *r*_*G*_ between VSC_q_ with the remaining traits were lower, ranging from 0.26 ± 0.56 (TNB) to 0.28 ± 0.41 (TND). For Farm3, moderate to high *r*_*G*_ estimates were found between VSC_q_ with AFS (− 0.32 ± 0.16), TNB (0.39 ± 0.26), NBA (0.31 ± 0.21), NS (0.69 ± 0.38), and SFS (− 0.71 ± 0.38). For the remaining traits, estimates were low, ranging from − 0.15 ± 0.14 (MUM) and 0.14 ± 0.39 (TND). Estimates of *r*_*P*_ were low between VSC_q_ with reproductive traits across all datasets. For Farm1, these ranged from − 0.07 ± 0.04 (SFS) to 0.04 ± 0.05 (AFS), for Farm2 from − 0.03 ± 0.04 (AFS) to 0.04 ± 0.04 (NSB), whereas for Farm3 these ranged from − 0.05 ± 0.02 (SFS) to 0.06 ± 0.02 (NBA). The efficiency of correlated response to selection when selecting for increased VSC, would be 1.80 and 2.37 for TNB and NBA, respectively, for across farms, 1.58 and 0.37 for TNB and NBA, respectively, for Farm1; 0.93 and 0.57 for TNB and NBA, respectively, for Farm3; and 0.25 for TNB for Farm2.

**Table 5:** Estimates of phenotypic (*r*_*P*_) and genetic (*r*_*G*_) correlations between vulva score categories^a^ and reproductive traits^b^ for each dataset and across datasets

Trait	Farm1 + 2 + 3		Farm1		Farm2		Farm3	
	*r*_*P*_	*r*_*G*_	*r*_*P*_	*r*_*G*_	*r*_*P*_	*r*_*G*_	*r*_*P*_	*r*_*G*_
AFS	0.05 (0.02)	0.25 (0.06)	0.04 (0.05)	0.17 (0.32)	−0.03 (0.04)	−0.51 (0.44)	0.01 (0.02)	−0.32 (0.16)
TNB	0.04 (0.02)	0.28 (0.19)	0.02 (0.04)	0.61 (0.47)	< 0.01 (0.04)	0.26 (0.56)	0.04 (0.02)	0.39 (0.26)
NBA	0.05 (0.02)	0.26 (0.17)	0.03 (0.04)	0.30 (0.37)	NC	NC	0.06 (0.02)	0.31 (0.21)
TND	−0.01 (0.02)	0.12 (0.20)	0.01 (0.04)	0.26 (0.51)	0.03 (0.04)	0.28 (0.41)	− 0.03 (0.02)	0.14 (0.39)
NSB	−0.01 (0.02)	0.01 (0.14)	−0.01 (0.04)	0.10 (0.55)	0.04 (0.04)	0.38 (0.35)	−0.01 (0.02)	0.13 (0.28)
MUM	−0.02 (0.02)	0.27 (0.79)	0.03 (0.04)	0.69 (0.47)	−0.03 (0.04)	−0.42 (0.58)	− 0.03 (0.02)	−0.15 (0.14)
NS	0.02 (0.02)	0.18 (0.13)	NC	NC	−0.02 (0.04)	−0.31 (0.59)	0.04 (0.02)	0.69 (0.38)
SFS	−0.06 (0.02)	−0.30 (0.13)	− 0.07 (0.04)	−0.36 (0.95)	0.03 (0.04)	0.33 (0.58)	−0.05 (0.02)	−0.71 (0.38)

^a^Vulva score categories as continuous variable (VSC_q_)

^b^*AFS* Age at first service, *TNB* Total number born, *NBA* Number born alive, *TND* Total number dead, *NSB* Number stillborn, *MUM* Number of mummies, *NS* Number of services until first farrow, *SFS* Unique service with successful first farrow

*NC* Not converged. Convergence was achieved when the REML log-likelihood and variance estimates change less than 0.002% and 1%, respectively, between consecutive iterations

### Genome-wide association analysis

**Fig. 1:**
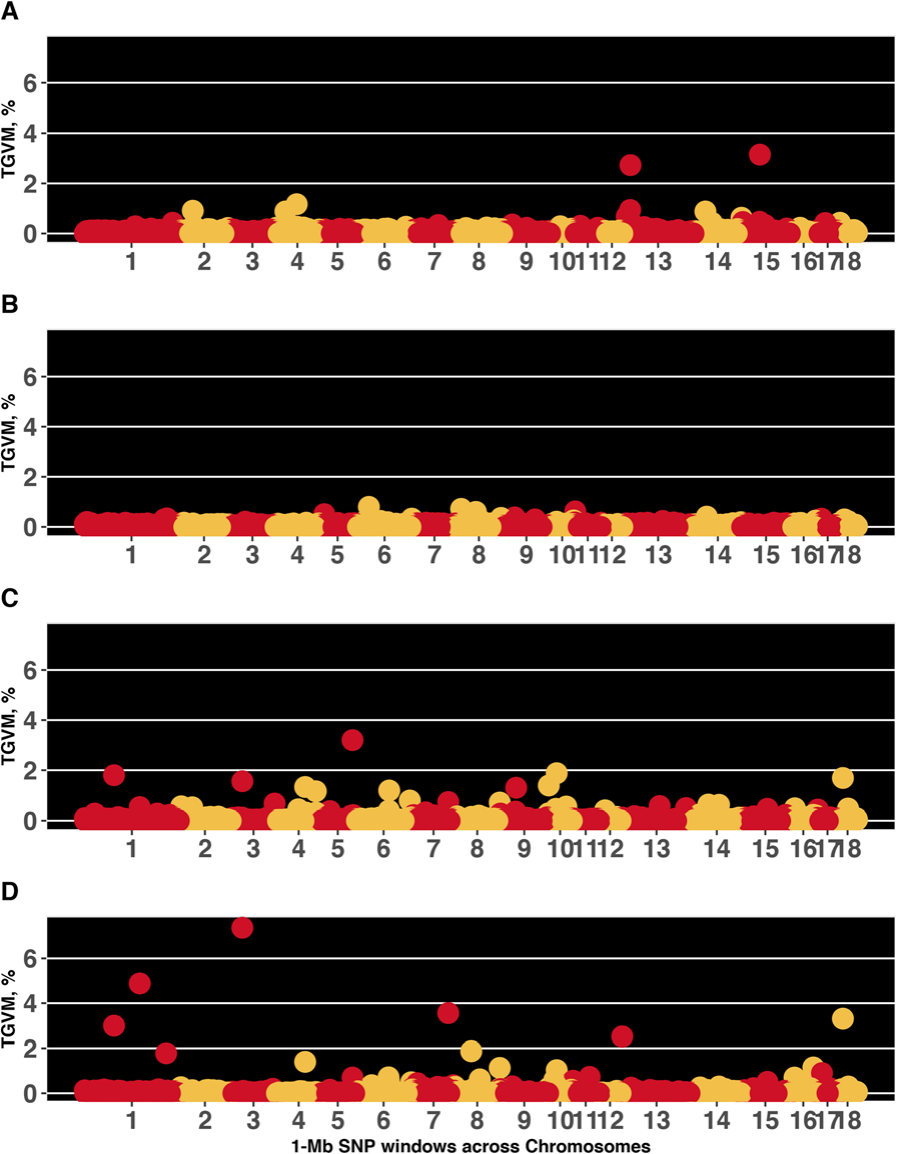
Manhattan plot for vulva score categories. Each data point represents a 1-Mb SNP window plotted against the percentage of total genetic variance accounted for by the markers (TGVM, %) in the window. The *X*-axis shows the chromosomes (1 to 18) of the 1-Mb SNP window, ordered according to their positions within chromosomes. Plots A, B, C, and D represent results for the datasets Farm1, Farm2, Farm3, and Farm1 + 2 + 3 respectively

Results from GWAS were similar for VSC_q_ and VSC_c_. Thus, only VSC_c_ results are presented in Fig. 1 and Table 6. A total of 20 unique genomic regions (quantitative trait loci; QTL) explaining more than 1% of the total genetic variance accounted for by the markers (TGVM) were identified across all analyses. For Farm1, 3 QTL were identified on SSC 4 (63 Mb), 13 (23 Mb), and 15 (52 Mb), explaining altogether 7.1%TGVM. For Farm2, no QTL explaining more than 1%TGVM were identified. For Farm3, 10 QTL were identified on SSC 1 (85 Mb), 3 (33–34 Mb), 4 (84 Mb and 115 Mb), 5 (91–99 Mb), 6 (102 Mb), 9 (46 Mb), 10 (1 Mb and 24–25 Mb), and 18 (14 Mb), explaining altogether 16.6%TGVM. Additionally, the GWAS including data from all farms identified 12 QTL, including SSC 1 (85 Mb), 3 (33–34 Mb), 4 (84 Mb), 10 (24–25 Mb), and 18 (14 Mb), and others not identified for the analysis using each farm separately: SSC 1 (160 Mb and 237 Mb), 7 (103–104 Mb), 8 (49 and 137 Mb), 13 (4 Mb), and 16 (69 Mb), altogether, these 12 regions explained 33.1%TGVM.

**Table 6:** Genomic regions associated with vulva score categories per dataset^a^

Dataset	SSC	Mb	No. of SNP	%TGVM
Farm1	4	63	26	1.2
	13	23	19	2.7
	15	52	15	3.2
Farm3	1	85	18	1.8
	3	33–34	54	1.6
	4	84	25	1.3
	4	115	24	1.2
	5	91–99	226	3.2
	6	102	21	1.2
	9	46	25	1.3
	10	1	26	1.4
	10	24–25	41	1.9
	18	14	31	1.7
Farms1 + 2 + 3	1	85	18	3.0
	1	160	21	4.9
	1	237	26	1.8
	3	33–34	54	7.4
	4	84	25	1.4
	7	103–104	44	3.6
	8	49	30	1.9
	8	137	15	1.1
	10	24–25	41	1.0
	13	4	30	2.5
	16	69	20	1.2
	18	14	31	3.3

*SSC Sus scrofa* chromosome, *Mb* Megabase location of the SNP window, *No. of SNP* Number of SNPs in the SNP window, *%TGVM* Total genetic variance accounted for by the markers

^a^There were no genomic regions explaining more than 1%TGVM for the Farm2 data

## Discussion

In this study, we investigated the relationship between VSC, assigned to 14- and 15-week old gilts, with subsequent reproductive performance. We aimed to corroborate, at both genetic and phenotypic levels, the previous findings from Graves et al. [2] and Romoser et al. [3]. Graves et al. [2] discovered a relationship (*r*_*P*_ = − 0.28, *P* = 0.01) between prepubertal vulva width and age at first estrus. These authors suggested that VSC measurements in gilts between 95 and 115 days of age could be used as a proxy for ovarian development and onset of puberty. Following this reasoning, Romoser et al. [3] determined that VSC-L gilts were more likely to achieve parity 1 compared to VSC-S (84.4% vs. 64.7%, respectively; *P* = 0.02), and presented greater TNB than VSC-S (12.4 vs. 11.8, respectively; *P* = 0.02). In addition to validating these results, we sought to explore the genomic basis of VSC in gilts.

### Phenotypic analysis of vulva score categories on reproductive traits

In general, significant phenotypic relationships between VSC and the traits evaluated were observed. However, the direction of the relationship (i.e. positive or negative) between VSC with these traits were not consistent. In Romoser et al. [3], there was a consistent relationship between VSC and reproductive traits, in which the greater was the VSC, the better was performance. Differently than in our study, these authors evaluated these relationships on the same farm. Furthermore, we fitted the random animal effect in the model used for these analyses, which was not the case for Romoser et al. [3]. This additional effect could have helped showing differences between both studies. Nonetheless, within a farm, results were overall reasonable. For example, in Farm1, there was a favorable relationship with NBA. Furthermore, the relationships between VSC and TNB were numerically positive, which is in accordance with the increased NBA observed in this farm. However, gilts with VSC-M and VSC-L had larger litter size but also larger TND, NSB and MUM, indicating that a greater VSC would increase overall litter size, in the expense of having dead piglets. For Farm2, sows with VSC-M and VSC-L had lower TNB but also lower TND and NSB. Furthermore, the relationships between VSC and MUM were favorably negative, which is in accordance with the decrease in TND. For Farm3, sows with VSC-M and VSC-L had higher TNB and NBA. Furthermore, the relationships between VSC and TND, NSB, and MUM were favorable. Gilts with VSC-L had lower TND, NSB and MUM. In general, the positive favorable relationships between VSC with TNB, and NBA for Farm1 and Farm3, and the negative favorable relationships between VSC with TND, NSB, and MUM for Farm2 and Farm3, were consistent with those observed in the study of Romoser et al. [3].

**Fig. 2:**
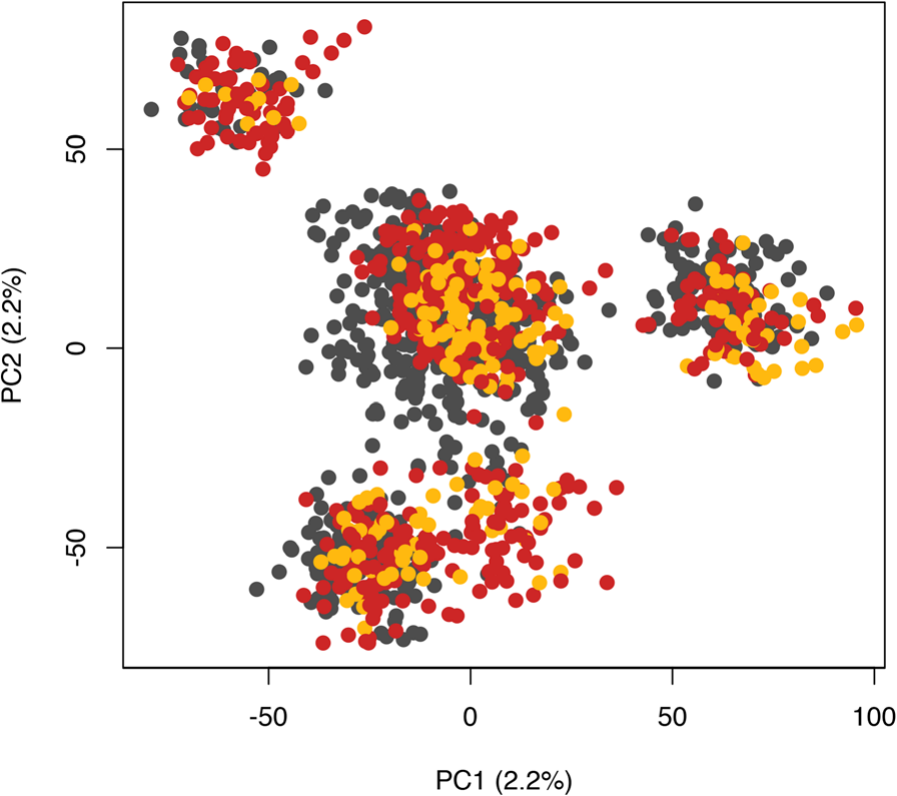
Population structure. Plot of first two principal components (PC2 and PC1) generated from SNP genotypes using the complete (Farm1 + 2 + 3) dataset. Each data point represents a single animal. Red dots represent animals from Farm1, yellow represent animals from Farm2, and grey represent animals from Farm3

The reasons for differences in VSC results between farms are unclear. In addition to the non-genetic factors that are inherited from each farm that do not allow us to separate them in our statistical analyses (i.e., confounded effects), two other processes could have resulted in these differences. First, the distributions of VSC-S in Farm1 and Farm2 were very different than in Farm3, with 2.8%, 1.7% and 21.2% in Farm1, Farm2, and Farm3, respectively. With this, the very low frequencies in Farm1 and Farm2 increased the standard error (SE) of the estimates, decreasing the statistical power to identify differences in performance based on VSC. This was not the case for Farm3. When ignoring the large SE, numerically, favorable phenotypic relationships were identified for TNB in Farm1, for MUM, NS, and SFS in Farm2, and for TND, NSB, and MUM in Farm3. Second, a different genetic makeup between farms could explain this. Animals in the three farms were from the same breed (Yorkshire) and were sourced from the same genetic source. In order to investigate the population structure of the three farms, we performed a principal component analysis (Fig. 2) with the use of the base *prcomp* function in R [15] . Although there were four visible clusters based on this analysis, we can see that the same clusters were formed across populations, indicating that they do share the same within population variation, but not between population variation. This could indicate that the differences in results should not be due to different genetic makeup of the three populations. With similar genetics and potential different environmental effects (which based on this data may not be additive), we could hypothesize that a possible explanation for the different results could be due to genotype-by-environmental interaction (i.e., G×E). If this is the case, proposing the use of VSC in pre-pubertal gilts as a selection tool for farrowing performance must be taken carefully, as the ideal environment must be obtained in order to identify this effect.

### Genetic parameters and efficiency of correlated response to selection

The heritability estimates across farms dataset and for each farm showed that VSC is highly heritable, and therefore, selection for this trait is possible. Heritability estimates for VSC_c_ and VSC_q_ estimates from the across farms dataset are similar than the ones reported for vulva width in crossbred Landrace × Large White gilts by Knauer et al. [4], with 0.57 ± 0.09, and for vulva size measurements by Corredor et al. [5], with 0.46 ± 0.10, 0.55 ± 0.10, and 0.31 ± 0.09 in Yorkshire population for vulva area, height, and width, respectively. However, these authors evaluated objective continuous VS measurements, whereas in the present study we only had categorical VS (VSC), which limited our power to properly link the observed variance with genetic variability in the population. In addition, the age difference during measurements between studies, with 23 and 14.5 weeks of age for Corredor et al. [5] and the current study, respectively, could be an added reason for the differences. Also, heritability estimates for VSC_q_ were estimated different than VSC_c_, however, they are not directly comparable since they are in different scales. Heritability estimates for VSC_q_ are in the scale of the observed data, while estimates of heritability for VSC_c_ were obtained with a non-linear (threshold) model and, therefore, the estimates are in the latent scale.

The estimates of genetic correlation for VSC_q_ between farms were overall high. The high estimate close to unity for Farm1 and Farm2 (0.97 ± 0.25) indicates that the animals with high genetic potential for VSC in one farm would have high genetic potential in the other farm. In fact, the estimates of genetic correlation between VSC_q_ and the reproductive traits for Farm1 and Farm2 had a similar direction and magnitude for TNB, TND, and NSB. This was also the case for Farm2 and Farm3, that had an estimate of genetic correlation close to unity (0.98 ± 0.16) and coincided with the direction of negative genetic correlations between VSC_q_ with AFS, and MUM, and positive genetic correlations between VSC_q_ with TNB, TND, and NSB. Between Farm1 and Farm3, we found a lower genetic correlation estimate (0.77 ± 0.22) for VSC_q_ compared to those for the other farms. This more moderate genetic correlation coincides with some greater differences in the direction of genetic correlations between VSC_q_ and the traits, such as for AFS and MUM. This moderate genetic correlation would indicate re-ranking of animals between Farm1 and Farm3, suggesting the presence of G×E.

In general, the heritability estimates for litter size traits TNB, NBA, TND, NSB, and MUM were low, and in accordance with recent reports in the literature [16–20], and thus, properly representing data from comparable studies. However, scarce literature is available for the genetic basis of other fertility related traits, such as AFS, NS, and SFS. Holm et al. [21], in a study with Landrace, reported heritability estimates for AFS and return rate on gilts (binary trait based on whether the gilt was re-inseminated after the first service), with 0.37 ± 0.01 and 0.03 ± 0.01, respectively. In our study, the heritability estimates for AFS in Farm1 was the same (0.37 ± 0.12) as in Holm et al. [21], whereas estimates for the other farms and across farms were numerically lower, with 0.27 ± 0.11, 0.14 ± 0.04, and 0.08 ± 0.03 for Farm2, Farm3, and across farms, respectively. In general, selection for improved AFS, NS, and SFS is feasible, and could result in more reproductively efficient sows.

Estimates of genetic correlations were positive between VSC_q_ with TNB, NBA, TND, and NSB across farms and for all three farms. These estimates indicated that a higher VSC corresponds genetically to larger TNB and NBA, further supporting the idea of using VS as a selection tool to increase NBA [3] but also high NSB and TND. However, the magnitude of these estimates differed across and between farms. Stronger favorable correlations between VSC with TNB and NBA were obtained in across farms, Farm1, and Farm3, which also had greater heritability estimate for VSC, compared to Farm2. In contrast, for the unfavorable correlations (TND and NSB), numerically, Farm2 had greater estimates. Even though there were unfavorable genetic correlation for VSC, the stronger favorable genetic correlations for TNB and NBA indicates that, overall, there is an overall benefit in selecting for increased VSC in Farm1 and Farm2.

There were contrasting genetic correlation estimates between VSC with AFS, MUM, NS, and SFS, depending on the dataset analyzed. For across farms and Farm1, all directions were unfavorable, whereas for Farm2, all directions were favorable, and for Farm3, unfavorable for NS and SFS, and favorable for AFS and MUM. The reasons for these differences are unknown. Finally, in general, many of these estimates had moderate to large standard errors, and hence, the value of the estimates should be taken carefully.

Given the favorable *r*_*G*_ estimates, the correlated response to selection for TNB and NBA would be limited. Although overall results indicate superior response to correlated response to selection for these traits using VSC_q_, the within farm analysis indicate that a greater efficiency would only be possible based on the results for Farm1. However, although there seems to be a limited impact of VSC on reproductive traits at the genetic level, there was a clear impact of VSC on reproductive traits at the phenotypic level (Farm1 and Farm3), indicating that there is great potential in using VSC for culling criterion. Regardless on this limitation, a combined phenotypic culling and genetic selection strategy could potentially be used to optimize selection for increased NBA in purebred populations. Selecting for VSC instead of a reproductive trait would be beneficial to anticipate selection, since VSC can be measured in gilts prior to insemination. In addition, this would allow for a higher intensity of selection because a larger population of gilts would be available prior to insemination. This advantage of having an early-age indicator of future reproductive performance could also increase response to selection by reduction the generation interval in female pigs. Thus, the use of VSC for both phenotypic and genetic purposes may be beneficial to the swine industry.

Finally, as seen for the phenotypic relationship between VSC with reproductive performance, results were somewhat different among the different datasets analyzed, even if for most cases the direction of correlations were similar across them. But, this additional inconsistency in results between farms suggest that 1) relationships within dataset might be real (similar results within datasets), which supports the hypothesis of 2) occurrence of G×E due to the non-genetic differences previously discussed in this study.

### Genome-wide association study

Results from genomic analyses differed among farms, which is in accordance with all other results presented in this study, further suggesting that non-genetic effects may be playing a role in the expression of VSC phenotypes between the three farms. Although there were no genomic regions identified for Farm2, Farm1 and Farm3 had associations with greater %TGVM for VSC than when the whole dataset was used for analysis (i.e., Farm1 + 2 + 3). However, analysis using Farm1 + 2 + 3 noted additional regions not identified when analyses were performed for each farm separately. This difference in results could be due to the much larger sample size used for the Farm1 + 2 + 3 (1083 observations compared to 193, 202, and 688, for Farm1, Farm2, and Farm3, respectively) which should have improved the statistical power to identify these additional QTL. Nonetheless, it is important to note that none of the Farm1 QTL were identified using Farm1 + 2 + 3. Given that the sample size of both farms was similar, the QTL identified in both Farm1 + 2 + 3 and Farm3 analyses could potentially indicate general QTL for VSC, whereas those identified in Farm1 or Farm3 and not in Farm1 + 2 + 3 could represent the potential occurrence of GxE in this study.

While Corredor et al. [5] investigated the genomic basis of quantitative vulva measurements in an independent dataset with Yorkshire and Landrace gilts, this is the first study to investigate the genomic basis of vulva qualitative assessments. Three QTL identified using data from Farm3 coincided with Corredor et al. [5]. In our study, the QTL identified on SSC 1 (85 Mb) is in close proximity to the one reported by Corredor et al. [5] on SSC 1 (87–91 Mb) as well as the one on SSC 10 (1 and 24–25 Mb) is in close proximity to the one reported on SSC 10 (8–19 Mb) by these authors for vulva area and height in Landrace gilts. For Farm1 + 2 + 3, some of the uniquely identified QTL also coincided with those reported by Corredor et al. [5]. The QTL on SSC 7 (103–104 Mb) is close to the one reported by these authors on SSC 7 (107–110 Mb) for vulva area and height in Landrace gilts.

Candidate genes related to reproductive development and performance were proposed for the identified regions that explained more than 3%TGVM, including the regions on SSC 1 (85 Mb and 160 Mb), 3 (33–34 Mb), 5 (91–99 Mb), 7 (103–104 Mb), 15 (52 Mb), and 18 (14 Mb). Within the QTL region on SSC 1 (85 Mb) serine protease 35 (*PRSS35*) is located, a gene that has been identified as a novel mouse ovary gene [22, 23]. Miyakoshi et al. [22] determined, using real-time polymerase chain reaction, that *PRSS35* was highly expressed at the time of ovulation and remained elevated in the developing corpus luteum. Wahlberg et al. [23] performed a study to identify new proteases that are involved in ovulation, using a microarray analysis of gene expression. Wahlberg et al. [23] found that *PRSS35* was highly expressed in the theca layers of developing follicles, and it was also expressed in the forming and regressing corpus luteum. Taken together, Miyakoshi et al. [22] and Wahlberg et al. [23] results suggested that *PRSS35* may be involved in ovulation in mice. In a study in humans, Li et al. [24] assessed the expression of *PRSS35* and observed that expression in cumulus cells of fertilized oocytes were significantly higher than those in cumulus cells of unfertilized oocytes. Li et al. [24] concluded that *PRSS35* may be correlated with oocyte fertility potential.

The QTL on SSC 1 (160 Mb) harbors serpin family B member 11 (*SERPINB11*). Yang et al. [25] investigated the expression of *SERPINB11* in mice uteri during early pregnancy and suggested that *SERPINB11* is involved in embryo implantation and decidualization. Similarly, Yang et al. [26] investigated the expression of *SERPINB11* in mice testis and suggested that *SERPINB11* might be involved in spermatogenesis. Likewise, Lim et al. [27] evaluated the expression profile of this gene across various tissues in chickens and observed high abundance of *SERPINB11* expression in the chicken oviduct, specifically in the luminal and glandular epithelia.

The gene protamine 1 (*PRM1*) is located within the QTL region on SSC 3 (33–34 Mb), which has been associated with sperm quality and embryonic early development in humans and pigs [28–32]. Depa-Martynów et al. [32] investigated the relationship between *PRM1* mRNA expression, among other genes, with embryonic development and sperm capacitation in humans. These authors concluded that *PRM1* mRNA expression could be used for estimating quality of spermatozoa in humans. However, this relationship has not been demonstrated in pigs [31].

Within the QTL region SSC 5 (91–99 Mb) we found KIT ligand (*KITLG*). In humans, *KITLG* has been associated with male infertility [33] and oocyte growth and follicular development [34, 35]. In porcine, expression of *KITLG* in the porcine ovary of prepuberal and mature animals by in situ hybridization showed that this gene is expressed in the granulosa cell layer and in the endothelial tissue and throughout the corpus luteum [36]. Brankin et al. [36] suggested that in the mature animal *KITLG* have a role in maintaining progesterone secretion by the corpus luteum.

The gene thyroid stimulating hormone receptor (*TSHR*) is located within the QTL region on SSC 7 (103–104 Mb). *TSHR* is a vital element in the pituitary thyroid axis of all vertebrates. *TSHR* commands to intracellular processes required for the synthesis, storage, and secretion of thyroid hormones, the main regulators of cellular metabolism [37, 38]. Karlsson et al. [39] investigating a domestic related mutation in the *TSHR*, found that it modulates photoperiodic response in chickens. These authors suggested that *TSHR* plays a key role in the signal transduction of seasonal reproduction. Rodríguez-Castelán et al. [40] explored the distribution of *TSHR* in reproductive organs of female rabbits. They found a presence of *TSHR* in the primordial, primary, secondary, tertiary, and Graafian follicles of virgin rabbits, as well as in the corpora lutea, corpora albicans, and wall of hemorrhagic cysts of pregnant rabbits. These wide presence of *TSHR* in female reproductive organs could suggests varied effects of *TSHR* in the reproduction of rabbits.

Neuregulin 1 (*NRG1*) resides within the QTL region on SSC 15 (52 Mb). Studies in mice and chickens have concluded that *NRG1* exerts an important regulatory role in oocyte meiotic maturation [41–43]. Jeon et al. [42] revealed that relative expression of *NRG1* mRNA increased in the oviducts of chicks treated with a synthetic non-steroidal estrogen. Furthermore, these authors suggested that *NRG1* is a novel estrogen-responsive gene closely correlated with the development of the oviduct of chicks.

The genes aldo-keto reductase family 1 member B (*AKR1B1*) and stimulated by retinoic acid 8 (*STRA8*) are located within the QTL region on SSC 18 (14 Mb). Multiple gene expression studies in humans and cattle demonstrated that *AKR1B1* is strongly associated with prostaglandin production, which is an important regulator of female reproductive function [44–48]. In pigs, *AKR1B1* functions in prostaglandin metabolism during the estrous cycle and pregnancy [49]. Studies in mice showed that *STRA8* expression is required for meiotic initiation in both female and male germ cells [50–53]. This gene has been explored in a study with transgenic pigs, observing the expression of this gene in testicular tissue [52]. These authors concluded that the expression of *STRA8* in transgenic pigs, from mouse *STRA8* promoter, could be useful as an animal model to study male germ cell manipulation and development.

In general, the genomic regions identified in this study for VSC include relevant genes for reproduction-related traits. Most of the genomic regions identified to be associated with VSC were associated with follicular and/or embryonic development. Furthermore, the genetic and phenotypic associations discovered between VSC and reproductive traits in this study additionally corroborate with the biologically relevant findings from our genomic analyses for VSC. Additional research to validate the use of VSC in different environments, using additional parities and genetic lines to continues assessment as an indicator trait of reproductive performance are warranted.

## Conclusion

In this study, phenotypic analyses support that VSC is associated with improved reproductive performance of sows, with large VSC gilts having greater NBA than small VSC gilts. VSC had moderate heritability among farms, showing that selection for VSC is possible. However, the genetic correlation for VSC between farm indicated the presence of G×E. For each farm, VSC was positively genetically correlated with TNB and NBA indicating that selection for greater VSC could result in increased litter size. Several genomic regions associated with VSC were identified, locating relevant candidate genes with reproductive function. These results support phenotypic relationship between VSC with TNB and NBA but that environmental factors could influence this relationship.

## Data Availability

The data that support the findings of this study are available from the breeding company, but restrictions apply to the availability of these data, which were used under license for the current study, and so are not publicly available. Data are however available from the authors upon reasonable request and with permission of the breeding company.

## Abbreviations

AFS: Age at first service
AKR1B1: Aldo-keto reductase family 1 member B
CG: Contemporary group
DNA: Deoxyribonucleic acid
E: Efficiency of correlated response to selection
Farm1: Farm 1
Farm2: Farm 2
Farm3: Farm 3
GWAS: Genome wide association study
GxE: Genotype-by-environmental interaction
*h*^2^: Heritability
KITLG: KIT ligand
ln: Natural logarithm
Mb: Megabase
MUM: Number of mummified piglets
NBA: Number of piglets born alive
NRG1: Neuregulin 1 gene
NS: Number of services until first farrow
NSB: Number of stillborn piglets
PRM1: Protamine 1
PRSS35: Serine protease 35
QTL: Quantitative trait loci
*r*_*G*_: Genetic correlation
*r*_*P*_: Estimates of phenotypic correlations
SE: Standard error
SERPINB11: Serpin family B member 11
SFS: Unique service with successful first farrow
SNP: Single-nucleotide polymorphism
SSC: *Sus scrofa* chromosome
STRA8: Stimulated by retinoic acid 8
TGVM: Total genetic variance accounted for by the markers
TNB: Total number of born piglets
TND: Total number of born dead piglets
TSHR: Thyroid stimulating hormone receptor
VSC-L: Vulva score category large
VSC-M: Vulva score category medium
VSC-S: Vulva score category small
VSC: Vulva score categories
VSC_c_: Vulva score as a categorical trait
VSC_q_: Vulva score as a continuous trait
